# Differential expression and regulation of HSP70 gene during growth phase in ruminants in response to heat stress

**DOI:** 10.1038/s41598-022-22728-6

**Published:** 2022-10-31

**Authors:** Rakesh Kaushik, Anjana Goel, P. K. Rout

**Affiliations:** 1 Department of Biotechnology, GLA University, 17Km Stone, NH-2, Mathura-Delhi Road, Chaumuhan, Mathura, 281406 U.P India; 2 Animal Genetics and Breeding Division, ICAR-Central Institute for Research on Goats, Makhdoom, Farah, Mathura, 281122 U.P India

**Keywords:** Biological techniques, Genetics, Molecular biology, Climate sciences, Biomarkers

## Abstract

Heat shock proteins regulate the physiological mechanism of heat stress adaptation at cellular level. The present investigation was carried out to analyse the HSP70 gene regulation in various growth stage in ruminants in peripheral blood mononuclear cells (PBMCs). The relationship between HSP gene expression and thermotolerance in age-specific manner in ruminants has not been analysed. Therefore m-RNA HSP70 expression level was examined in different age groups of Jamunpari goat during hot climatic conditions. The experiment was carried out in 32 animals of Jamunapari goat belonging to the age groups of 3-months, 9-months, 12-months, and adults (2–3 year). Total RNA was isolated from peripheral blood mononuclear cells. The physiological response such as rectal temperature (RT), respiration rate (RR) and heart rate (HR) was used as indicator to heat stress. Temperature Humidity Index (THI) was used as an indicator of severity of environmental stress. The THI range varied from 82.00–92.08 during experimental period. The m-RNA HSP70 expression level at 9-month age of animals was up-regulated and significantly higher than other age groups. It was observed that the level of HSP70 transcripts in PBMCs was highest at 9-month age group, and age-related decline in HSP70 expression was observed in adult age. Based on the physiological response, the contrasting heat-stress phenotypes were recognised as heat stress susceptible (HSS) and heat stress tolerant (HST) individuals and the expression of m-RNA HSP70 was analysed at different ages in response to chronic heat stress. The differential mRNA expression of HSS individuals at 3 and 9-month of age showed the highest fold expression than HST. Age and phenotype had significant effect (*p* < 0.01) on the crossing point (CP) value. The m-RNA HSP70 gene expression in different age groups was correlated with heat stress tolerance and this could be used as biomarker for breeders to analyse the HSP response *in -vivo* in ruminants.

## Introduction

Thermal acclimation and thermal adaptation are associated with increased basal level of heat shock proteins (HSPs)^1,2^. HSPs are activated in response to various environmental and other stressors. It has been observed that the skin epithelium releases heat shock protein to mobilize the thermal shock in response to heat stress. The expression of inducible HSP70 is increased several-fold as skin temperature approaches the upper limit of the thermo-neutral zone of ruminants. The heat stress regulation pathway protects the proteome of all the cells in response to elevated temperatures, and oxidative damage^3,4^. At the cellular level, heat and other metabolic stressors induce the HSPs and increase gene expression due to the activation of heat shock transcription factors (HSFs)^5–7^. HSPs interact with other cellular proteins during stress conditions and maintain cellular homeostasis^8^. The individual animals respond to physiological and environmental stress by activating different stress regulation pathways which protect the core biological processes by promoting protein folding. HSPs are released intracellularly and extracellularly in an inducible form in response to stress. Cellular tolerance to heat stress is regulated by HSPs and HSP70 can be an indicator of stress in cells^9^. HSPs are responsible for maintaining the balance between survival and an effective immune system in the organism in order to acclimatize the stress^10^.

The ability to withstand heat stress is an important component of adult fitness. Cells release heat shock proteins in response to metabolic or environmental stresses^8^. It has been observed that there was a decline in the heat-induced expression of HSP 70 m-RNA in primary fibroblast of rat with ageing^11^. Similarly, the decline in the heat-induced expression of HSP70 in human diploid fibroblasts as a function of cell passage (in vitro ageing) has been reported. The age-dependent thermoregulation at physiological level has been observed in human as well as experimental animals^12^. It has been also shown that HSP regulates stress tolerance at tissue level in vivo^13^. Therefore, the present study was carried out to analyse the age-related m-RNA HSP70 expression in response to heat stress in ruminants. The study was carried out in vivo to observe the mechanism of thermoregulation in animals up to 1 year of age and adult individuals during heat stress period. HSP 70 m-RNA expression was analysed during growth phase of ruminants till maturity age and in adult animals in response to heat stress. The regulation of HSP70 expression with respect to the aging process in vivo has not been carried out in ruminants. The differential expression of HSP70 protein is still not analyzed and understood in ruminants, therefore the present study analyses the expression pattern of heat shock protein at different ages during heat stress period.

## Results

The environmental temperature during the experimental period varied from 40 to 49.5 °C. Temperature-humidity index (THI) varied from 82.0–92.08 during hot period. The physiological response such as rectal temperature (RT), respiration rate (RR) and heart rate (HR) exhibited wide variability in animals during heat stress period and are presented in Table 1. The range of variability in RT, RR and HR was 38.1 to 39.9 °C, 24 to 84 breaths/min, and 100 to 160 beats/min during the hot period, respectively (Table 1). The classification of phenotypic differences at the population level was based on the earlier presented data. On the basis of the distribution of RR and HR, individuals having a RR of ≤ 34 (breaths/min) and a HR of ≤ 108 (beats/min) were recognized as heat stress-tolerant phenotype (HST). However, RR of ≥ 50 (breaths/min) and a HR of ≥ 130 (beats/min) were recognized as heat stress-susceptible phenotype (HSS). The mean variation of physiological responses during extreme heat stress period with respect to stress susceptible phenotype is presented in Table 2 and Supplementary Fig. 1. The mean of RT, RR and HR showed significant variation within heat stress susceptible and tolerant phenotype. The mean of RT, RR and HR of heat susceptible phenotype were 39.277 °C, 70.923breaths/min and 145.538 beats/min, respectively. The mean of heat stress susceptible phenotype and tolerant differenced by 0.859 °C in RT, 39.650 breaths/min in RR and 37.083 beats/min in HR, respectively. The mean variation of physiological responses in different ages with respect to HSS and HST stress phenotype are presented in Table 3. The RT, RR and HR were significantly (*P* < 0.01) different between HSS and HST phenotypes at different age groups. Therefore, RT, RR, and HR were significantly (*P* < 0.01) affected by age of the animals.

**Table 1:** Mean environmental conditions and physiological responses (RT, RR and HR) during heat stress period.

Environmental conditions		
Parameters	Mean ± SE	Range
Duration (days)	28	
Temperature (°C)	45.946 ± 0.518	40.0–49.5
RH (%)	28.203 ± 1.763	14.33–51.00
Rainfall (mm)	0.128 ± 0.076	0.0–1.80
Sunshine (h)	10.071 ± 0.313	4.80–12.00
THI	88.506 ± 0.493	82.00–92.08
RR	52.075 ± 5.089	24–84
HR	128.541 ± 4.030	100–160
RT	38.883 ± 0.141	38.1–39.9

*RH* Relative humidity in percentage; *THI* Temperature-humidity index; *°C* Degree centigrade; % Percentage; *RR* Respiration rate (breaths/min); *HR* Heart Rate (beats/min); *RT* Rectal temperature in degree centigrade (°C); *SE* Standard Error of mean; *mm* millimeters; *h* hours.

**Table 2:** Mean of physiological responses (RT, RR and HR) in heat stress-tolerant and heat stress-susceptible phenotype in Jamunapari goats during heat stress period.

Phenotype	No. of observations	RT (°C)	RR (breaths/min)	HR (beats/min)
Heat stress tolerant (HST)	16	38.418 ± 0.202^a^	31.273 ± 1.329^a^	108.455 ± 1.371^a^
Heat stress susceptible (HSS)	16	39.277 ± 0.116^b^	70.923 ± 3.808^b^	145.538 ± 1.897^b^

*No* Number or sample size; *RT* Rectal temperature in degree centigrade (°C); *RR* Respiration rate (breaths/per min); *HR* Heart rate (beats/per min), *Means* ± *SE* means within the same rows with different superscripts are significantly different (*p* < 0.01).

**Table 3:** Means of physiological responses (RT, RR and HR) of different age groups in heat stress-tolerant and susceptible phenotype during the heat stress period.

Age group	Phenotype	No. of observations	HR	RR	RT
3 M	HST	04	113 ± 1.17^a^	34 ± 1.16^a^	39.20 ± 0.29^a^
	HSS	04	156 ± 2.31^b^	100.50 ± 0.29^b^	39.75 ± 0.09^a^
9 M	HST	04	102 ± 1.16^a^	26 ± 1.15^a^	39.30 ± 0.06^a^
	HSS	04	135 ± 2.89^b^	65 ± 8.66^b^	39.75 ± 0.03^b^
12 M	HST	04	104 ± 2.31^a^	26 ± 1.15^a^	38.65 ± 0.32^a^
	HSS	04	142 ± 1.16^b^	65 ± 8.66^b^	39.55 ± 0.09^b^
Adults	HST	04	107.50 ± 0.29^a^	34 ± 1.16^a^	39.15 ± 0.03^a^
	HSS	04	142 ± 1.16^b^	84 ± 2.31^b^	39.60 ± 0.06^b^

*No* Number or sample size; *3 M* 3-Month of age; *9 M* 9-month of age; *12 M* 12-month of age; Adults, 2–3 year age of animals; *HST* Heat stress tolerant; *HSS* Heat stress susceptible; *RR* Respiration rate (breaths/min); *HR* Heart Rate (beats/min); *RT* Rectal temperature in degree centigrade (°C). *Means* ± *SE* Means ± Standard error, means within the same rows with different superscripts are significantly different (*p* < 0.01).

### HSP 70 m-RNA expression in growing kids and adult individuals

**Figure 1:**
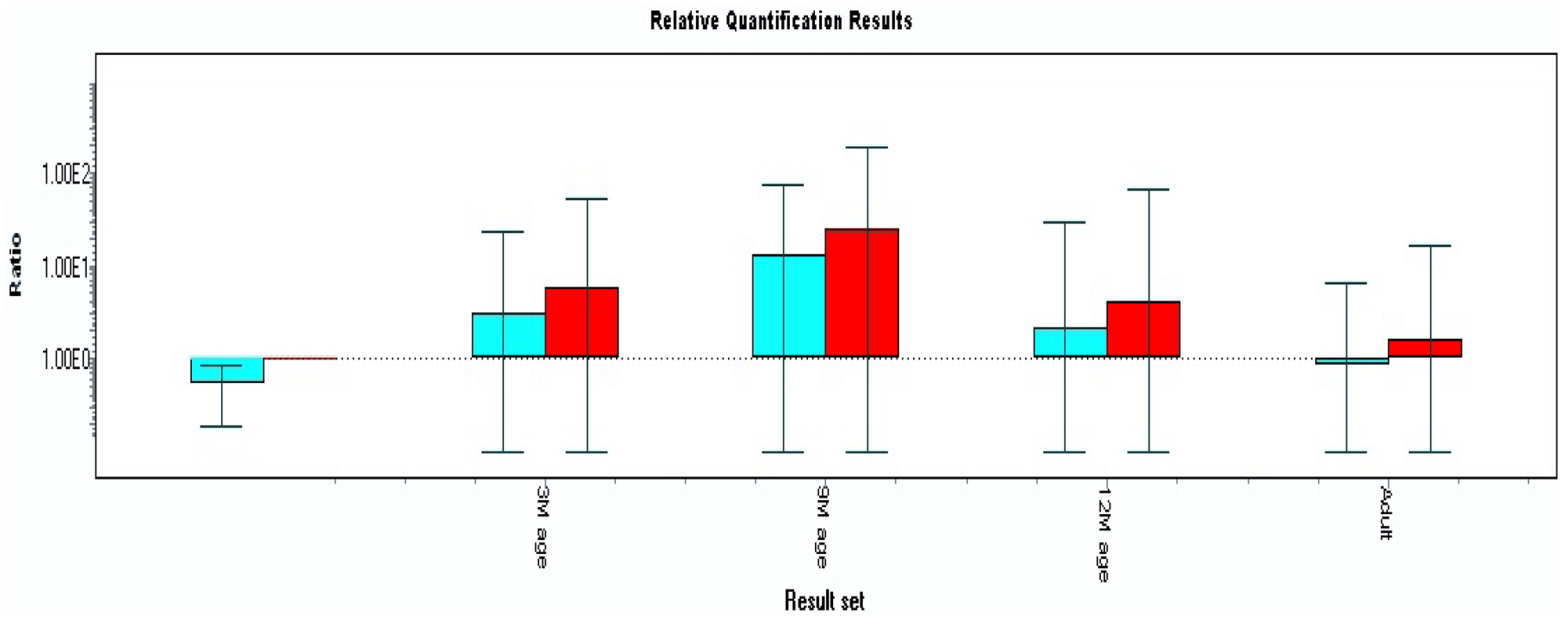
Relative mRNA expression (fold change) of HSP70 gene in different age groups of Jamunapari goat. 3 M, 3-month age of animal; 9 M, 9-month age of animal; 12 M, 12-month age of animal; adult, 2–3 year age of animals. The crossing point (Cp) readings for each unknown sample were then used to calculate the amount of either the target or housekeeping gene using the second derivative maximum method with the Light cycler 480 analysis software version 1.5 (Roche Applied Science, Indianapolis, IL, USA). GADPH and β-actin were used to normalize gene expression. The susceptible individual was used as a positive calibrator to obtain normalized gene expression.

The relative m-RNA expression level of HSP70 was analysed in the different age groups of Jamunapari goats during hot period. The m-RNA expression pattern of HSP70 was analysed at 3, 9, 12 months of age and in adults (2–3 years) with respect to stress phenotypes. Glyceraldehyde 3-phosphate dehydrogenase (GAPDH) and beta actin (*β*-actin) genes were used as internal control. Quality of amplified product by using RT-PCR (Supplementary Fig. 2), amplification curve & melting peak are provided in Supplementary Fig. 3A and Fig. 3B. The relative m-RNA expression pattern of HSP70 gene in 3, 9, 12 months and adults ages showed 5.70, 23.90, 4.08 and 1.51 up-regulation compared to adult control (heat stress-susceptible) (Table 4 and Fig. 1). The m-RNA expression was significantly higher at 9-months of age of animals as compared to other age groups. The expression of HSP70 gene at 9-month age of the Jamunapari goats was 23.9-fold higher in comparison to calibrator (control group). In addition, the 9 months of age showed a 5.86-fold and 15.83-fold higher m-RNA level than the 12-month of age and adult, respectively (Table 4 and Fig. 1).

**Table 4:** Relative mRNA expression (fold change) of HSP70 gene in different age groups of Jamunapari goats.

Age	No.of observations	Target/Reference	Relative fold Expression
Calibrator (control)-adult	4	0.53	1
3 month	8	3.03	5.70
9 month	8	12.72	23.90
12 month	8	2.17	4.08
Adult	4	0.80	1.51

*No.* Number or sample size; 3, 9, 12 month and adult, Age of Jamunapari goats.

**Figure 2:**
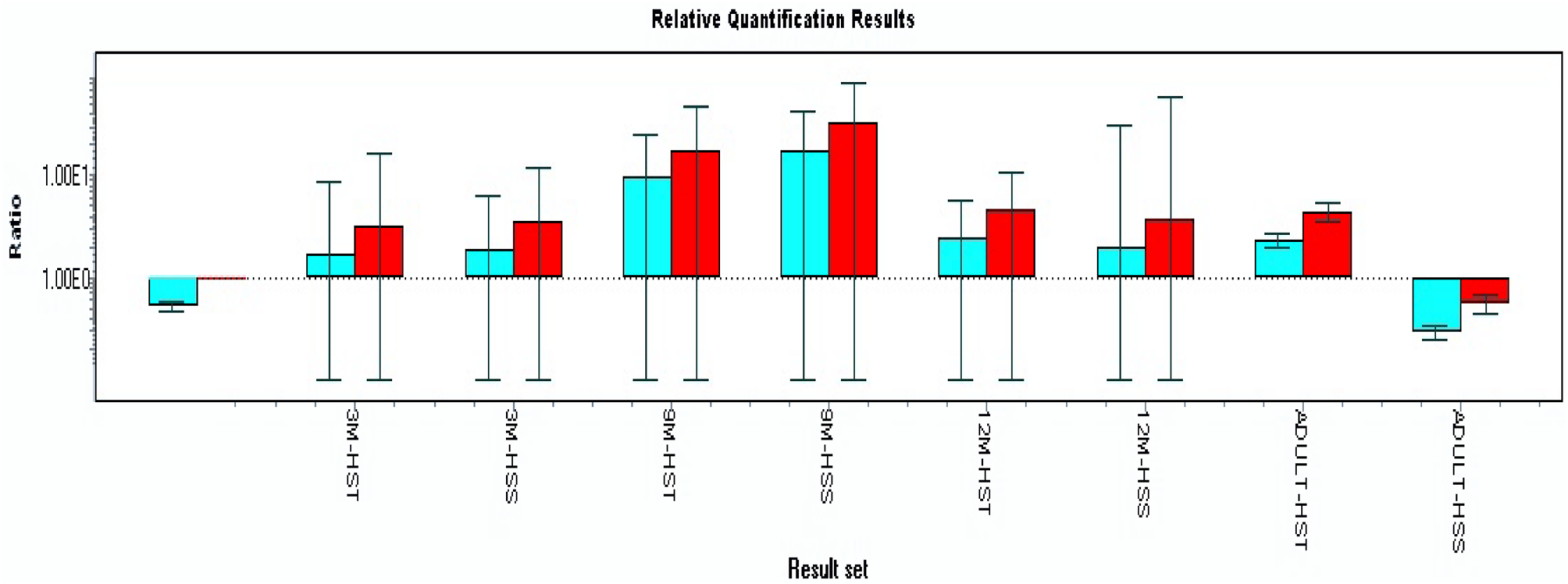
Relative mRNA expression (Fold change) of HSP70 gene at different age groups of Jamunapari goat with respect to HST and HSS phenotype. 3 M-HST, 3-Month age of animal- Heat stress tolerant phenotype; 3 M-HSS, 3-Month age of animal-Heat stress susceptible phenotype; 9 M-HST, 9-Month age of animal-Heat stress tolerant phenotype; 9 M-HSS, 9-Month age of animal-Heat stress susceptible phenotype; 12 M-HST, 12-Month age of animal-Heat stress tolerant phenotype; 12 M-HSS, 12-Month age of animal-Heat stress susceptible phenotype; Adult-HST, Adult (2–3 year) age of animal- Heat stress tolerant phenotype; Adult-HSS, Adult 92–3 year) age of animal- Heat stress susceptible phenotype. The crossing point (Cp) readings for each unknown sample were then used to calculate the amount of either the target or housekeeping gene using the second derivative maximum method with the Light cycler 480 analysis software version 1.5 (Roche Applied Science, Indianapolis, IL, USA). GADPH and β-actin were used to normalize gene expression. The susceptible individual was used as a positive calibrator to obtain normalized gene expression.

However, the differential m-RNA expression between contrasting heat stress susceptible and tolerant phenotypes indicated that HSS individuals at 3, 9, 12 months and adult ages exhibited 3.57, 32.34, 3.70 and 0.54-fold higher expression than control. Similarly, HST individuals at 3, 9, 12 months and adult exhibited 3.20, 17.66, 4.50 and 4.41- fold higher expression than control. The m-RNA expression of HSP70 gene based on heat stress phenotypes were 3.57 and 32.34- fold higher in heat stress- susceptible phenotype at 3 and 9-month age of animals, respectively. Similarly, the heat stress-tolerant phenotype exhibited 3.20 and 17.66-fold lower expression at 3 and 9-month age during the hot period (Table 5 and Fig. 2).

**Table 5:** Relative mRNA expression (Fold change) of HSP70 gene at different age groups of Jamunapari goat with respect to HST and HSS phenotype.

Phenotype	Age	No. of observations	Target / Reference	Relative Fold Expression
Heat stress susceptible (HSS)	Calibrator (control)-adult	4	0.53	1
	3 month	4	1.90	3.57 ± 8.18
	9 month	4	17.22	32.34 ± 45.98
	12 month	4	1.97	3.70 ± 56.34
	Adult	2	0.28	0.54 ± 0.10
Heat stress tolerance (HST)	3 month	4	1.70	3.20 ± 13.48
	9 month	4	9.40	17.66 ± 30.06
	12 month	4	2.39	4.50 ± 5.95
	Adult	2	2.35	4.41 ± 0.89

*No.* Number or sample size; 3, 9, 12 month and adult, Age of Jamunapari goats.

The least squares mean of crossing point (CP) value were analysed in the different age groups of Jamunapari goats. Table 6 shows the effect of season of birth, birth type, sex, age, phenotype and parity. HSS phenotype showed significantly higher (*p* < 0.01) CP than HST phenotype. Sex by phenotype interaction showed a significant difference (*p* < 0.01) on CP (Table 7). However, there was no significant difference observed in the season of birth, birth type and sex on CP. The ANOVA table revealed that age, phenotype, and parity had significant effect (*p* < 0.01) on CP (Table 8). Similarly, season of birth, birth type, age and parity had significant (*p* < 0.01) effect on body weight.

**Table 6:** The least squares means showing the effect on crossing point value (CP) in Jamunapari goats.

Factor	Sample size	CP
**Age**		
3 month	8	29.64 ± 0.32^a^
9 month	8	29.45 ± 0.34^a^
12 month	8	30.59 ± 0.29^b^
Adults	8	30.66 ± 0.22^b^
**Phenotype**		
HST	16	29.83 ± 0.15^a^
HSS	16	30.35 ± 0.20^b^
**Parity**		
1	8	29.58 ± 0.22^a^
2	8	30.62 ± 0.30^b^
3	8	29.78 ± 0.25^ab^
4	8	30.36 ± 0.18^b^

*CP* Crossing point value; *HST* Heat-stress tolerant phenotype; *HSS* Heat-stress-susceptible phenotype; *Means* ± *SE* Means ± Standard Error, means within the same rows with different superscripts are significantly different (*p* < 0.01).

**Table 7:** The least squares mean interaction on crossing point value (CP) between sex by phenotype.

Interaction sex by phenotype		
Factor	No. of observations	CP
Male-HST	6	28.95 ± 0.39^a^
Male-HSS	2	30.37 ± 0.26^ab^
Female-HST	10	30.72 ± 0.19^b^
Female-HSS	14	29.82 ± 0.09^c^

*No* Number or sample size; *CP* Crossing point value; *Male-HST* Male-Heat stress tolerant phenotype; *Male-HSS* Male-Heat stress susceptible phenotype; *Female-HST* Female-Heat stress-tolerant phenotype; *Female-HSS* Female-Heat stress susceptible phenotype; *Means* ± *SE* Means ± Standard Error, means within the same rows with different superscripts are significantly different (*p* < 0.01).

**Table 8:** ANOVA of HSP70 showing the effect of genetic and non-genetic factors on crossing point (CP) values and body weight in Jamunapari goats.

Source		CP			Body Weight		
	Df	MSS	F	P	MSS	F	P
Season of birth	1	0.017	0.114	NS	4.507	10.862	**
Birth type	1	0.150	0.993	NS	1.307	3.149	*
Sex	1	0.030	0.202	NS	131.600	317.184	***
Age	3	0.974	6.443	**	140.669	339.041	***
Phenotype	1	0.970	6.420	*	0.148	0.356	NS
Parity	3	1.047	6.927	**	0.656	1.580	NS
Age x phenotype	3	0.590	7.529	**	48.994	118.087	***
Sex x phenotype	1	1.464	18.662	**	67.797	163.404	***

*Df* Degree of freedom; *MSS* Mean Sum of squares; *F* F-value; *P* ***P < 0.001, **P < 0.01, *P < 0.05; *NS* Non-significant; *CP* Crossing point; Season of birth, February–March and October–November.

Birth type: Single & Multiple; Age, 3, 9, 12 months and adults age of animals; Phenotype–Heat stress-tolerant (HST) and Heat stress-susceptible (HSS); Parity, Parity of dam; Age x phenotype, Interaction between age and phenotype; Sex x phenotype, Interaction between sex and phenotype.

## Discussion

HSP 70 plays a protective role during heat stress and also regulates normal cell growth and proliferation. HSPs protect cells against apoptosis mediated through oxidative stress^24^. Cellular thermal stress tolerance is regulated by HSPs^25–27^ and HSP70 is considered as a marker for heat stress tolerance in different species^28,29^. The age-related heat-stress regulation and thermal-tolerance in hot environmental conditions in livestock have not been well understood. The m-RNA expression pattern of HSP70 showed that HSP70 gene expression was significantly higher at 9-month age as compared to 12 months and adult age in Jamunapari goats. The expression of m-RNA HSP70 was about 5.85 and 15.75 folds higher as compared to 12-months and adult age. The expression level of m-RNA HSP70 gene in 3, 9, 12-months and adults ages showed 5.70, 23.90, 4.082 and 1.51 up-regulation compared to adult control (heat stress- susceptible individuals). Growth and development in goats occur in a similar way compared to other ruminants and mammals. Growth is a function of the life cycle of each animal that begins with embryo fertilization and ends with death. Cells are the basic unit of growth and development. Growth and developmental rates are governed by both genetic potential and environmental factors. The foetal phase of growth is from differentiation to parturition. The factors that affect post-natal growth and development are genetic potential and the influence of environment and nutrition to attain the genetic make-up. The goats attain maturity during 9 months of age and it is the age for maturation of the immune system. As it is evident the maternal immunity affects the individual up to 6 months of age. Therefore 9-month is the age the age for selecting individual for growth and other economic traits. Sexual maturity is the age at which mammals can reproduce and attains development in every phase. Rodent family attains sexual maturity at the age of 1–2-month, Dog and Bovidae family reach sexual maturity at about 1 year of age and primate including human being reaches maturity at the age of 23 years^30^. Growth and development are determined by single and interactive multiple factors of the external and internal environments. It is required to understand the growth and development in goats and the factors that affect these processes as it determines the efficiency of production and product quality. The rate and efficiency of growth and the subsequent effects on product quality need to be manipulated from conception to consumption for better human health and effective resource management^31^. Exploration of expression patterns and its relevance in survival, and adaptation holds a great promise in livestock improvement and breeding regimens.

Similarly, the m-RNA expression of HSP70 at 3, 9, 12 and adults were 3.57, 32.34, 3.70 and 0.54-fold in HSS phenotype and 3.20, 17.66, 4.50 and 4.41- fold in HST phenotype, respectively. The differential m-RNA expression indicated that the HSS individuals at 3 and 9 months of age had highest fold expression than HST. Similarly, m-RNA gene expression profile study for HSP60 and HSP70 has also been reported in Saanen goats^32^. In addition, a significantly positive correlation of HSP70 and 60 was observed between environmental condition and physiological parameters in dairy goats^33^. The seasonal profile of HSP60 and 70 concentrations have been reported to be less in winter season as compared to summer and found a positive and significant correlation between HSP concentration and physiological data in goats^34^. In this study, the animals of 1–2-year-old (youngest group) showed highest increase in HSP expression as compared 3–4 year and 5–6-year-old individuals. Moreover, a seasonal variation was also recorded with HSP70 level was found with elevated HSP60 and HSP70 expression during the summer as compared to other seasons. These findings are also consistent with smaller mammals, especially rodents, such as male Wistar rats^11^. It has been established that there was a decline in the heat-induced expression of HSP70 m-RNA in primary fibroblast of rat with ageing ^11^. Also, HSP70 expression was 40–50 percent lower in adult rats (22–28 months) than in young rats (4–7 months) when exposed to 42.5 °C for 30 min^35^. In the present study, physiological responses indicated significant variation within heat stress- susceptible and tolerant phenotype. Similarly, HSP70 on serum level was associated with some physiological parameters in dairy goats under south turkey conditions ^36^. In *Drosophila melanogaster* age-dependent and sex-dependent expression of HSP70, HSP22, and HSF1 was studied and observed that HSP70 expression declines throughout the life-span. HSP induction is important in maintaining homeostasis, then a deficit in its expression could contribute to an age-related decrease in stress tolerance. Alternations in Hypothalamus-Pituitary Axis (HPA) function are known to occur with age^37–43^. Although it has been reported that there is no age-related deficit in eliciting an adrenocortical response to acute stress, it has been suggested that reduced HPA activity occurs in aged animals after repeated stress exposure^44^. Thus, the decline in HSP70 expression with age could reflect a change in HPA activity rather than an intrinsic alternation in HSP70 gene regulation. HSP70 appears to play a protective role to cope up in these stress conditions and may function to protect cells against subsequent challenges^13,45,46^.

The regulation of HSP70 gene expression is complex. Similarly, the decline in the heat-induced expression of HSP70 in human diploid fibroblasts as a function of cell passage (in vitro ageing). The transcriptional mechanism of HSP70 varies in relation to age and it may be due to age-associated alteration in the signaling mechanism of heat shock response^47^. The age-dependent thermoregulation at physiological level has been observed in humans as well as experimental animals^12^. In a preliminary study using human peripheral blood mononuclear cells, a 30% impedance of heat-induced HSP70-encoding gene transcription was observed in aged persons relative to young individuals^48^. In human, serum Hsp70 was positively correlated with age within 30 years and negatively correlated with Hsp70 level in lymphocyte after 40 years^49^. The present results could improve our understanding of the mechanism of thermotolerance in the ruminants during growth phase and factors affecting growth and economic traits. Therefore, it is necessary to analyse the correlation between m-RNA expression and protein expression in different age groups during heat stress period. Similarly, it is also required to evaluate serum protein of the animal and determine heat shock balance index (eHsp70/ iHsp70) in particular population for better adaptability and maintain productivity in the changing climatic variation.

## Conclusion

The m-RNA HSP70 expression level at 9-month age of animals was up-regulated than other age groups. HSP70 m-RNA levels were higher in HST individuals at 3 and 9-months of age of Jamunapari goat. The age of 9 months is the age for selecting individuals for growth and other economic traits. The m-RNA HSP70 gene expression in different age groups was correlated with heat stress tolerance and this could be used as biomarker for breeders to analyse the HSP response in vivo in ruminants.

## Materials and methods

The experiment was carried out at the Jamunapari breeding unit at ICAR-Central Institute for Research on Goats (ICAR-CIRG), Makhdoom, Mathura, Uttar Pradesh, India. Jamunapari is one of the most milk-producing and large sized goat breeds in India, distributed in the semi-arid region of Uttar Pradesh^14^. The climate in the study area was semi-arid, with average temperature of 45 °C and precipitation of ~ 400 mm during the experimental period. Animals (goats) were housed separately based on their gender, age, health and physiological status, and were managed under semi-intensive rearing system with 6–7 h grazing time. Appropriate animal feed, including dry fodder and green fodder, were provided based as per physiological and production status. The body condition score was adequate and uniform for all the animals. At regular intervals, the flock was vaccinated and dewormed.

### Animal selection and physiological responses

The investigation has been carried out in Jamunapari goat breed of semi-arid region of India, and categorised into four age groups, as shown in Table 9. In growing kids, physiological responses such as respiration rate (RR), heart rate (HR), and rectal temperature (RT) were recorded as indicators of heat stress. Physiological responses were recorded during the highest temperature of the day ranging from 13.30 to 14.30 h. The physiological response at various ages was recorded three times over 8–10-day period (May–June). A digital clinical thermometer was used to measure rectal temperature (accuracy ± 0.1 °C). RR and HR were measured by auscultation as described earlier ^15^.

**Table 9:** Age of Jamunapari goats along with number of animals and coat color.

Age groups	No. of animals	Coat color
3-months age	08	White color, tall, roman nose, pendulous ears/large-sized
9-months age	08	
12-months age	08	
Adult (2–3 year) age	08	

### Recordings of meteorological data and temperature humidity index (THI)

The data on meteorological variables (relative humidity (%), sunshine (h), rainfall (mm), dry bulb temperature (DBT) and wet bulb temperature (WBT) and temperature) were recorded at ICAR- Central Institute for Research on Goats, Makhdoom, Farah, Mathura. The THI was calculated from dry and wet bulb air temperatures for a particular day according to the following formula:

$${\text{THI}} = \, 0.{72 }\;\left( {{\text{DBT}} + {\text{WBT}}} \right) \, + {4}0.{6}$$

where, dry and wet bulbs are temperature in degrees Celsius. The collection and recording of physiological responses with respect to highest THI during the peak heat stress period varied from 82.00–92.08. THI could be used to predict thermal climatic conditions^16^. Extreme heat stress period, the average of environmental temperature ranged from 40.0–49.5 °C and RH ranged from 14.33–51.0 and animals were exposed to radiation for 4–5 h for 28 days.

### Selection of heat stress-tolerant and susceptible phenotype

To distinguish the two contrasting phenotypes, the distribution of high respiration rate and heart rate and low respiration rate and heart rate was used. In goats, respiration activity and heart rate serve as indicators of heat stress tolerance, and individuals are classified as heat stress-tolerant or susceptible. The phenotypic classification of heat stress in goats has been extensively described at the population level elsewhere ^17–21^.

### Animal, sampling and isolation of peripheral blood mononuclear cells (PBMC)

About 3–4 ml fresh blood samples from each animal was collected aseptically in heparinized vacutainer tubes (BD Biosciences, Franklin Lakes, NJ, USA) through jugular vein puncture and immediately transported under refrigeration for the isolation of RNA. The ethical guidelines were followed during blood collection. The blood samples were diluted with PBS, pH 7.4 (1:2) and subsequently layered upon volume of HiSep LSM-1077(Hi-Media). The precautions were taken to produce a clean interface between two layers of blood and HiSep media. Samples were centrifuged at 3000 rpm at 4 °C for 30 min and the white opaque mononuclear fraction of cells from the interface was aspirated into fresh micro-centrifuge tubes (MCT). Diethyl Pyrocarbonate (DEPC)-Phosphate Buffer Saline (DPBS) was added to resuspend the caprine PBMCs and further centrifuged for washing at 5000 rpm for 5 min. Finally, the obtained cell pellet was transferred to a sterile DEPC treated micro-centrifuge tube.

### RNA isolation, cDNA synthesis and m-RNA expression analysis

Total RNA was isolated from PBMCs using TRIzol (Invitrogen) method. One milliliter of TRIzol was re-suspended to dissolve the PBMCs pellet. Subsequently, RNA isolation method was followed according to Rout and Kaushik et al. ^17,20^. The quality and quantity of RNA were assessed by Biophotometer (Eppendorf) by using OD260 for concentration and the ratios 260/280 and 260/230 to assess the purity of the sample. The RNA integrity was tested on a 1.4% agarose gel, and samples that passed the purity test (A280 ~ 1.9) were used for cDNA synthesis. 1 µg of RNA was used for the preparation of cDNA by transcriptor first strand cDNA synthesis kit (Roche) following by manufacturer’s protocol, and the cDNA thus obtained was stored at − 70 °C for future use. DNase treatment was used to remove DNA contamination from RNA, as previously described^17^. Real Time PCR was analysis was carried out in Light Cycler 480 (Roche Applied Science, Indianapolis, IL, USA) using SYBR Green® master mix (Roche) as per manufacturer instructions. The reaction was set up in 96 well plate and each well contained 2 μL of cDNA sample, 10 μL of SYBR green I master mix (Roche Applied Science, Indianapolis, IL, USA), 1 μL (20 pmol) of the specific primers and nuclease-free water to make final volume of 20 μL. The primers of HSP70 gene (5′ TCATCGGAGATGCAGCCAAGAA-3′ and R-5′ AGATCTCCTCGGGGAAGAAGGT 3′) were used with an annealing temperature of 61 °C to amplify a 210 bp fragment. GAPDH (F-5′ GTGATGCTGGTGCTGAGTAC3′ and R-5′ GTAGAAGAGTGAGTGTCGC-3′) and β-Actin (F-5′ TGCCCT GAGGCTCTCTTCCA′ and R- 5′ TGCGGATGTCGACGTCACA-3) were used to normalise the gene expression of the HSP70 gene.

The thermal profile was standardized as initial denaturation at 94 °C for 10 min, followed by 45 cycles, denaturation at 94 °C for 10 s, annealing at 61 °C for 15 s, and extension at 72 °C for 20 s. The tests were carried out in duplicate. PCR products were subjected to melting curve analysis in the Light cycler 480 and subsequently 3% agarose gel electrophoresis to confirm amplification specificity and amplicon size.

### Relative expression analysis

Relative quantification was carried out to measure fold-change in expression levels of the target genes using the 2^–∆∆Ct^ method and by E-method^22^. The advance relative quantification was carried out using the second derivative maximum method with the Light Cycler 480 analysis software version 1.5 (Roche Applied Science, Indianapolis, IL, USA). All analyses were performed on mean Cp value, which were calculated from two sample replicates used in real-time PCR.

### Statistical analysis

The expression and physiological responses within stress phenotypes were analysed. For fitting constants, mixed model least-squares means analysis was used to determine the statistically significant effect of various genetic and non-genetic factors^23^. The model includes the fixed effect of season of birth (2 levels), birth type (2 levels), sex (2 levels), age (4 levels), phenotype (2 levels), parity (4 levels) and interaction effect. The crossing point (CP) of HSP70 gene and body weight of Jamunapari goat was fitted as a linear covariate in the model.

Model 1:

$${\text{Y}}_{{{\text{ijklmn}} = }} {\upmu } + {\text{ Season of birth}}_{{\text{i}}} + {\text{ Birth type}}_{{{\text{j }} + }} {\text{Sex}}_{{{\text{k }} + }} {\text{Age}}_{{\text{l}}} + {\text{ Phenotype}}_{{\text{m}}} + {\text{ Parity}}_{{{\text{n }} + }} \left( {{\text{Age}} \times {\text{Phenotype}}} \right)_{{{\text{lm}}}}+\left( {{\text{Sex}} \times {\text{Phenotype}}} \right)_{{{\text{km}}}}$$

where, Y_ijklmn_ is the observation of *i*th season of birth, *j*th birth type, *k*th sex, *l*th age group, *m*th phenotype, *n*th parity, µ = population of mean, Season of birth = fixed effect of *i*th season of birth (February–March and October–November = 1 and 2), Birth type_j_ = fixed effect of *j*th birth type (Single and Twins, J = 1 and 2), Sex_k_ = fixed effect of *k*th sex (Male and Female, K = 1 and 2), Age_l_ = fixed effect of *l*th age group (3-month, 9-month, 12-month and adults l = 1, 2, 3 and 4), Phenotype_m_ = fixed effect of *mth* phenotype (Heat stress-tolerant and Heat stress-susceptible, m = 1 and 2), Parity_n_ = fixed effect of *n*th  parity (Parity = 1 to 4), Eijklmn = random residual error associated with observation with mean 0 & variance 1.

### Ethical approval

All sample collection was conducted in accordance with institutional practice and the study was approved by Institutional animal ethics committee (IAEC/CIRG/18–19). Norms of arrive were followed during the ethical approval process.

## Supplementary Information

Supplementary Figures.

## Data Availability

The datasets used and/or analysed during the current study available from the corresponding author on reasonable request.

## References

[1] Carper SW, Duffy JJ, Gerner EW (1987). Heat shock proteins in thermotolerance and other cellular processes. Can. Res..

[2] Kregel KC (2002). Invited review—Heat shock proteins: Modifying factors in physiological stress responses and acquired thermotolerance. J. Appl. Physiol..

[3] Calderwood SK, Murshid A, Prince T (2009). The shock of aging: Molecular chaperones and the heat shock response in longevity and aging—A mini-review. Gerontology.

[4] Kim B, Park K, Rhee K (2013). Heat stress response of male germ cells. Cell. Mol. Life Sci..

[5] Sorger PK (1987). The transcriptional regulation of heat shock genes (Doctoral dissertation).

[6] Sorger PK, Lewis MJ, Pelham HR (1987). Heat shock factor is regulated differently in yeast and HeLa cells. Nature.

[7] Zimarino V, Wu C (1987). Induction of sequence-specific binding of Drosophila heat shock activator protein without protein synthesis. Nature.

[8] Lindquist S, Craig EA (1988). The heat-shock proteins. Annu. Rev. Genet.

[9] Sonna LA, Fujita J, Gaffin SL, Lilly CM (2002). Invited review: Effects of heat and cold stress on mammalian gene expression. J. Appl. Physiol..

[10] Morange F (2006). HSFs in development. Handb. Exp. Pharmacol..

[11] Fargnoli J, Kunisada T, Fornace AJ, Schneider EL, Holbrook NJ (1990). Decreased expression of heat shock protein 70 mRNA and protein after heat treatment in cells of aged rats. Proc. Natl. Acad. Sci..

[12] Finch, C. E., Landfield, P. W. in *Handbook of the Biology of Aging* (eds. Finch, C. E. & Schneider, E. L.) (Van Nostrand Reinhold, New York, 1978) pp. 567–594.

[13] Barbe MF, Tytell M, Gower DJ, Welch WJ (1988). Hyperthermia protects against light damage in the rat retina. Science.

[14] Rout PK, Kaushik R, Ramachandran N, Jindal SK (2017). Identification of heat stress-susceptible and-tolerant phenotypes in goats in semiarid tropics. Animal Production Science.

[15] Andrews AH, Blood DC, Radostits OM (1988). Veterinary medicine. A textbook of the Diseases of Cattle Sheep Pigs Goats and Horses.

[16] McDowell RE, Hooven NW, Camoens JK (1976). Effect of climate on performance of Holsteins in first lactation. J. Dairy Sci..

[17] Rout PK, Kaushik R, Ramachandran N (2016). Differential expression pattern of heat shock protein 70 gene in tissues and heat stress phenotypes in goats during peak heat stress period. Cell Stress Chaperones.

[18] Kaushik R, Dige MS, Rout PK (2016). Molecular characterization and expression profiling of ENOX2 gene in response to heat stress in goats. Cell Dev. Biol.

[19] Kaushik R, Dige M, Dass G, Ramachandran N, Rout PK (2018). Superoxide dismutase activity in response to heat stress in Jamunapari goats. Indian J. Biochem. Biophys..

[20] Kaushik R, Goel A, Rout PK (2019). Differential expression and characterization of ATP1A1 exon17 gene by high resolution melting analysis and RT-PCR in Indian goats. Mol. Biol. Rep..

[21] Kaushik R, Goel A, Rout PK (2020). Establishing the genetic variation in physiological response in response to heat stress in semi-arid region in Jamunapari goats. Biol. Rhythm. Res..

[22] Livak KJ, Schmittgen TD (2001). Analysis of relative gene expression data using real-time quantitative PCR and the 2^−ΔΔCT^ method. Methods.

[23] Harvey, W. R. User's Guide for LSMLMW In: *Mixed model least Squares and Maximum Likelihood Computer Program PC-Version*, Vol. 2, (Ohio State University Press, Ohio, 1990).

[24] Beere HM, Wolf BB, Cain K, Mosser DD, Mahboubi A, Kuwana T, Tailor P, Morimoto RI, Cohen GM, Green DR (2000). Heat-shock protein 70 inhibits apoptosis by preventing recruitment of procaspase-9 to the Apaf-1 apoptosome. Nat. Cell Biol..

[25] Sonna LA, Fujita J, Gaffin SL, Lilly CM (1985). Invited review: Effects of heat and cold stress on mammalian gene expression. J. Appl. Physiol..

[26] Hecker JG, Sundram H, Zou S, Praestgaard A, Bavaria JE, Ramchandren S, McGarvey M (2008). Heat shock proteins HSP70 and HSP27 in the cerebral spinal fluid of patients undergoing thoracic aneurysm repair correlate with the probability of postoperative paralysis. Cell Stress Chaperones.

[27] Hecker JG, McGarvey M (2011). Heat shock proteins as biomarkers for the rapid detection of brain and spinal cord ischemia: A review and comparison to other methods of detection in thoracic aneurysm repair. Cell Stress Chaperones.

[28] Archana PR, Aleena J, Pragna P, Vidya MK, Niyas APA, Bagath M, Krishnan G, Manimaran A, Beena V, Kurien EK, Sejian V (2017). Role of heat shock proteins in livestock adaptation to heat stress. J. Dairy Vet. Anim. Res..

[29] Hyder I, Pasumarti M, Reddy PR, Prasad CS, Kumar KA, Sejian V, Alexzander AA, Asea PK (2017). Thermotolerance in domestic ruminants: A HSP70 perspective. Heat Shock Proteins in Veterinary Medicine and Sciences.

[30] Pacifici M, Santini L, Di Marco M, Baisero D, Francucci L, Marasini GG, Visconti P, Rondinini C (2013). Generation length for mammals. Nat. Conserv..

[31] Webb EC, Casey NH (2010). Physiological limits to growth and the related effects on meat quality. Livest. Sci..

[32] Yilmaz M, Kayki M, Aşici G, Kiral F (2018). The mRNA gene expression profiles for HSP60 and HSP70 in various aged Saanen goats in different seasons. Kafkas Üniv. Vet. Fak. Derg..

[33] Alyamani, D., Koluman, N. (2019) Associated expressions of heat shock protein (70 and 60) with physiological adaptation with in dairy goats. Available at SSRN 3535801.

[34] Alyamani D (2020). Impact various seasons on expression patterns HSP60 and physiological parameters. J Dairy Vet Anim Res.

[35] Heydari AR, Wu B, Takahashi RYOYA, Strong RANDY, Richardson A (1993). Expression of heat shock protein 70 is altered by age and diet at the level of transcription. Mol. Cell. Biol..

[36] Yamani HAL, Koluman N (2020). Association HSP70 with some physiological parameters in dairy goat under south Turkey conditions. J. Dairy. Vet. Anim. Res..

[37] Pavlov EP, Harman SM, Chrousos GP, Loriaux DL, Blackman MR (1986). Responses of plasma adrenocorticotropin, cortisol, and dehydroepiandrosterone to ovine corticotropin-releasing hormone in healthy aging men. J. Clin. Endocrinol. Metab..

[38] Roth G (1976). Brain Res..

[39] Brodish A, Odio M (1989). Age-dependent effects of chronic stress on ACTH and corticosterone responses to an acute novel stress. Neuroendocrinology.

[40] Ottenweller JE, Tapp WN, Pitman DL, Natelson BH (1990). Interactions among the effects of aging, chronic disease, and stress on adrenocortical function in Syrian hamsters. Endocrinology.

[41] Landfield PW, Waymire JC, Lynch G (1978). Hippocampal aging and adrenocorticoids: Quantitative correlations. Science.

[42] Sapolsky RM, Krey LC, McEWEN BS (1983). Corticosterone receptors decline in a site-specific manner in the aged rat brain. Brain Res..

[43] Sapolsky RM, Krey LC, McEwen BS (1986). The neuroendocrinology of stress and aging: The glucocorticoid cascade hypothesis. Endocr. Rev..

[44] Odio M, Brodish A (1989). Age-related adaptation of pituitary-adrenocortical responses to stress. Neuroendocrinology.

[45] Finley D, Ciechanover A, Varshavsky A (1984). Thermolability of ubiquitin-activating enzyme from the mammalian cell cycle mutant ts85. Cell.

[46] Gershon H, Gershon D (1973). Inactive enzyme molecules in aging mice: Liver aldolase. Proc. Natl. Acad. Sci..

[47] Liu AY, Lin Z, Choi HS, Sorhage F, Li B (1989). Attenuated induction of heat shock gene expression in aging diploid fibroblasts. J. Biol. Chem..

[48] Deguchi Y, Negoro S, Kishimoto S (1998). Molecular chaperones and the aging process Biochem. Biophys. Res. Commun..

[49] Jin X, Wang R, Xiao C, Cheng L, Wang F, Yang L, Feng T, Chen M, Chen S, Fu X, Deng J (2004). Serum and lymphocyte levels of heat shock protein 70 in aging: A study in the normal Chinese population. Cell Stress Chaperones.

