# Supplementary material for: Differential expression and regulation of HSP70 gene during growth phase in ruminants in response to heat stress
Source: Sci Rep. 2022 Oct 31;12:18310. doi: 10.1038/s41598-022-22728-6 (PMC9622898; doi:10.1038/s41598-022-22728-6)
Supplement: Supplementary file 1 — Supplementary Figures. [file 41598_2022_22728_MOESM1_ESM.pdf]

Supplementary Figures

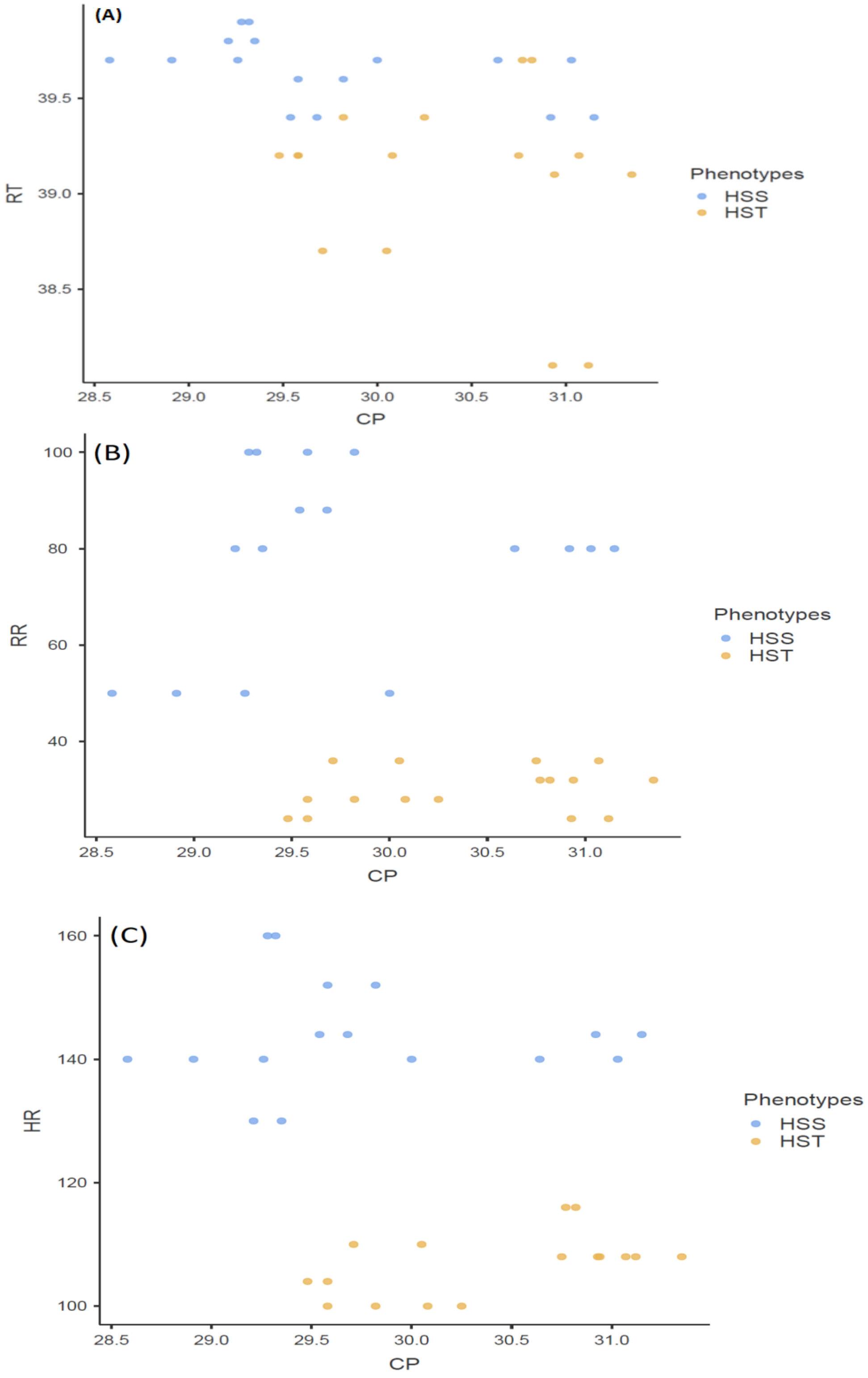

Figure S1. Phenotype comparison between Physiological responses (RT (A), RR (B), and HR(C) and crossing point values. RT, Rectal temperature in degree centigrade (°C); RR, Respiration rate

(breaths/min); HR, Heart rate (beats/min); CP, Crossing point value; HST, Heat stress- tolerant phenotype; HSS, Heat-stress-susceptible phenotype.

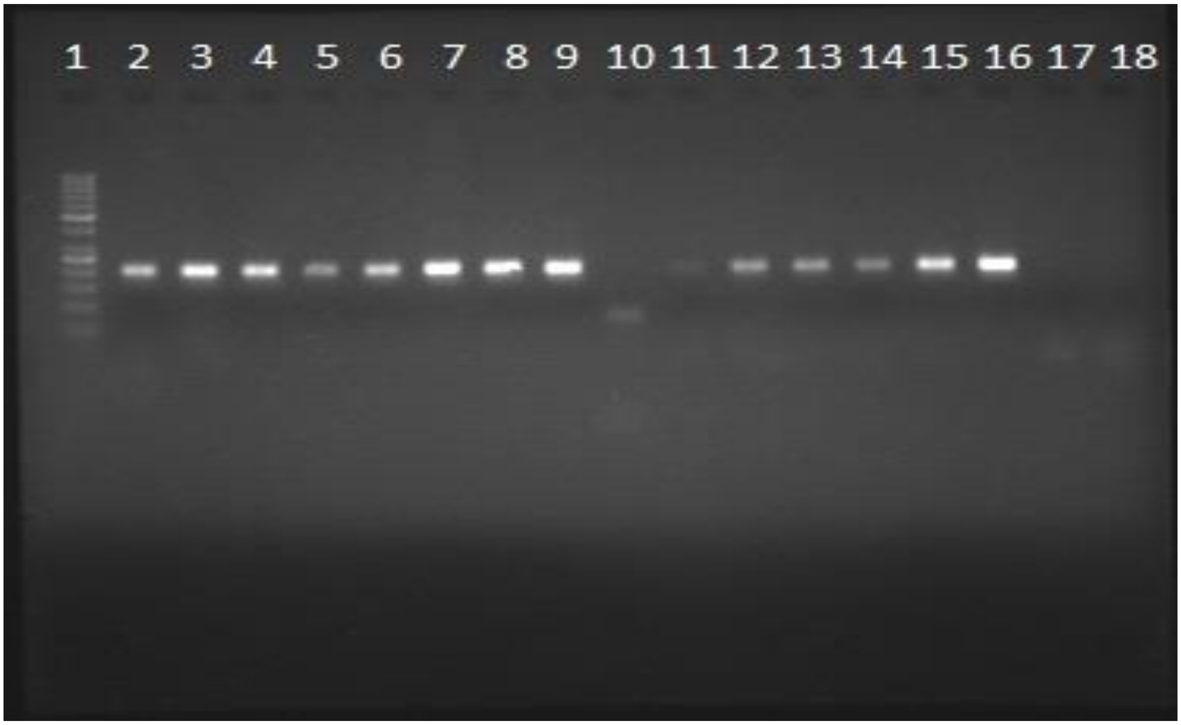

Figure S2. Agarose electrophoresis of amplified product of HSP70 by RT-PCR. Lane-1, 50bp (Marker); Lane-2-5, 3-Month age of animals; Lane 6-9, 9-Month age of animals; Lane-10, Negative control; Lane 11-14; 12-month of age; Lane-15, Adult (2-3 year) age; Lane-16; PCR Product; Lane-17-18, Blank.

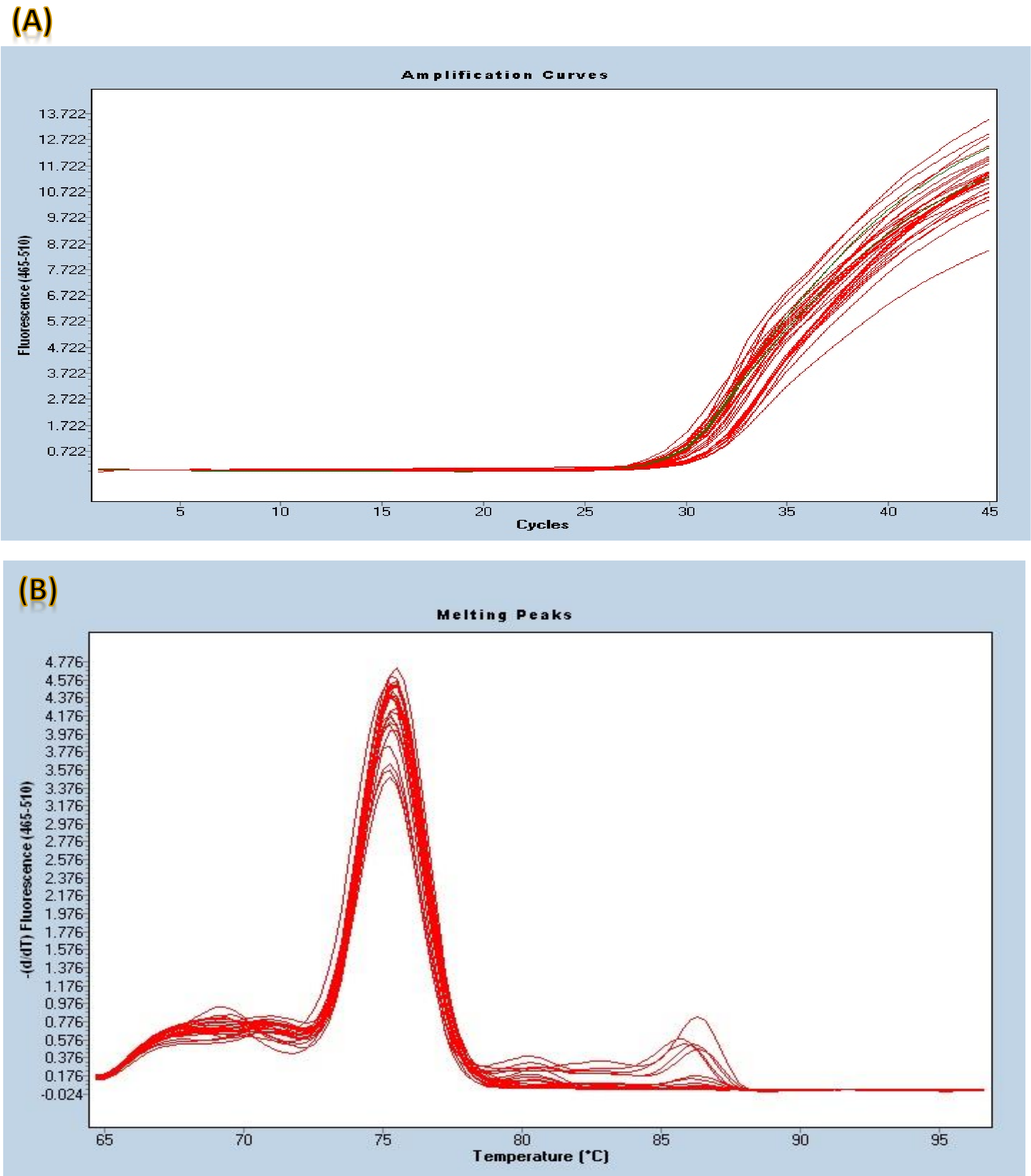

Figure S3. (A) Amplification curve and Melting curve **(B)** of HSP70 gene in Jamunapari goat at different age groups.
